# Emerging Diseases/Viruses Prevention, Control, Surveillance, and One Health

**DOI:** 10.3390/tropicalmed8050257

**Published:** 2023-04-29

**Authors:** Yannick Simonin

**Affiliations:** Pathogenesis and Control of Chronic and Emerging Infections, University of Montpellier, INSERM, Etablissement Français du San, 34394 Montpellier, France; yannick.simonin@umontpellier.fr

Emerging diseases have posed a constant threat and major challenge to human health throughout our history. From the Black Death in the Middle Ages to the COVID-19 pandemic, these diseases have generated significant human suffering and economic disruption. The emergence of new pathogens, such as Ebola, Zika, SARS-CoV-2 or mpox, has illustrated the potential for these diseases to spread extraordinarily rapidly. Far from dissipating despite remarkable advances in medical science and public health in recent decades, emerging diseases continue to appear and proliferate at an alarming rate. Changes in our environment, produced primarily by human activity and the evolution of human/animal interactions, are likely to be responsible for novel health crises in the future.

Therefore, prioritizing the prevention of emerging diseases is essential in order to prepare for these future health crises. Preventive measures for emerging diseases involve early detection and rapid responses, but also the development of effective vaccines and treatments. Strengthening surveillance systems and enhancing diagnostic capacities are essential in order to detect and respond rapidly to epidemics. In addition, it is vital to boost investment in public health infrastructure, particularly with regard to sanitation and hygiene in general, in order to mitigate the risk of disease transmission. In order to achieve this, public health systems must be equipped with the necessary resources and trained personnel to detect and respond rapidly to epidemics, which both require significant investment. In addition to preventive measures, control measures are required in order to contain emerging diseases. The SARS-CoV-2 pandemic has revealed to us that the contact tracing of patients and the isolation and quarantine of infected people, all coupled with extensive vaccination campaigns, are productive control measures. In addition, effective communication strategies are required in order to prevent emerging diseases and must be designed to reach all factions of the population, including isolated groups and those with limited access to health care, particularly those who live on the streets.

Prevention measures for emerging diseases must often be implemented on a global scale. This requires international cooperation and coordination to quickly detect and respond to outbreaks. A fundamental approach to strengthening surveillance systems is the One Health concept. This concept recognizes that human health, animal health and environmental health are interdependent. Zoonoses represent at least 60% of infectious diseases and no less than two-thirds of novel emerging diseases. Therefore, monitoring and containing animal populations can greatly aid in the prevention of novel pathogens emerging. Furthermore, monitoring environmental factors, such as temperature and humidity, may help anticipate the emergence of novel pathogens, including vector-borne diseases.

Amongst the pathogens that possess a significant potential for emergence are arboviruses, whose distribution is constantly expanding in correlation with their vectors, particularly mosquitoes. Indeed, climate change, urbanization and land use all affect the dynamics of vectors, as well as reservoir host populations and the transmission of vector-borne pathogens. In this context, the geographical distribution of arboviruses is expanding; it now affects all five continents and has become a major public health concern. Among the 26 articles collected in this SI, 10 articles attend to arboviruses. Dengue (DENV) is the most diffuse arbovirosis in the world, and DENV cases have been surging in recent years. Li et al. review the literature and reveal studies that have collected age-specific DENV serological data in China; they discover that the transmission intensity varies depending on age in most of the study populations, and the attenuation of antibody protection was identified in some study populations [1]. Melo et al. report the first pediatric disease in which the use of minimally invasive autopsy has confirmed severe dengue as the cause of death [2]. Chong et al., in a narrative review, detail the impact of DENV on pregnancy in Southeast Asia, revealing, in particular, the specific physiological effects of dengue during the trimesters of pregnancy [3]. Another study uses the combination of a DENV and zika virus (ZIKV) nonstructural protein 1 IgG enzyme-linked immunosorbent assay and a ZIKV NS1 blockade-of-binding ELISA in order to test the convalescent sera of non-flavivirus, primary DENV, secondary DENV, and ZIKV infections. The authors discover that primary testing via a ZIKV NS1 IgG ELISA is the prime option for large-scale ZIKV serosurvey studies and provides relatively high sensitivity [4]. Regarding WNV, one article of this SI details the detection of WNV lineage 2 infection in birds from the Umbria region during the cold season; it confirms that the L2 strains of WNV that circulate in Italy are genetically stable and provides evidence of a continuous circulation of WNV in Italy throughout the year [5]. Another study foregrounds the correlation between oxidation and severe disease in WNV-infected patients [6]. Mhamadi et al. illustrate the circulation of Rift Valley fever virus in northern Senegal through human and livestock surveillance [7], while Stevanovic et al. demonstrate the rather high seroprevalence of Tahyna orthobunyavirus, a neglected mosquito-borne bunyavirus, in human and animal (horses and pets) serum in a One Health approach; this evidences the criticality of studying this neglected arbovirus [8]. Another report studies the seroprevalence of DENV, ZIKV, or CHIKV in Bangphae District, Ratchaburi Province, Thailand, via enzyme-linked immunosorbent assays and rapid diagnostic tests [9]. Furthermore, one review details the role of CHIKV infection in arthritogenic pain, which appears to be comparable to rheumatoid arthritis as both diseases share common symptoms [10]. 

Five articles in this SI attend to the COVID-19 pandemic from various perspectives. De Carvalho et al. summarize the COVID-19 infodemic on Twitter in Brazil via a study that applies a thematic analysis across space and time with respect to public opinion regarding the Brazilian COVID-19 immunization program [11]. Another study highlights the influence of climatic and environmental elements on the proliferation of COVID-19 in Africa [12]. The research article by Zhang et al. proposes five models through which to study the early COVID-19 outbreak in Shaanxi, China. They demonstrate that the renewal equation model provides the optimum modelling and significantly enhances the estimate of transmissibility [13]. In a brief report, Fernández-Santos et al. suggest that vulnerable populations traveling from Latin American countries and seeking residence in the United States are at a high risk of exposure to SARS-CoV-2 [14]. Another study reveals that quarantine effectively diminishes the number of COVID-19 cases but induces an escalating number people suffering from tuberculosis and diabetes; this emphasizes the criticality of promoting a healthy lifestyle when implementing quarantine [15].

Two further articles attend to HIV; the first studies the barriers to and facilitators of adherence to antiretroviral therapy in Indonesia by employing a socioecological approach [16]. The second evaluates the impact of COVID-19 on receiving an HIV diagnosis in children, particularly regarding the prevention of the mother-to-child transmission of HIV in Johannesburg, South Africa [17]. 

This SI also includes four articles that focus on parasite-related issues and in particular, two articles on leishmaniasis. The first, from Israel, reveals that there is no tangible variation regarding sex difference in leishmanial infection in humans [18]. The second article provides novel evidence for the modification of differentially expressed circRNAs and their potential function in leishmaniasis [19]. Kim et al. compare the efficacies of the recombinant vaccinia viruses that express either the AMA1 or microneme protein (MIC) of *Plasmodium berghei* in mice. Their results indicate that the recombinant vaccinia viruses that express MIC could be an advantageous candidate vaccine- antigen [20]. Another study aims to analyze the prevalence and characteristics of malaria and influenza co-infection in febrile patients. The prevalence of this co-infection among these patients is revealed to be heterogeneous by country, the characteristics of the febrile participants, and the diagnostic tests for influenza virus [21].

Four further articles attend to bacterial infections. An article from Zhong et al. reveal that *Escherichia coli* ST1193 isolates have emerged as the predominant type of *E. coli* strain that causes intracranial infections in Changsha, China [22]. A case report by Safiee et al. indicates that the various infecting *Leptospira* species and the presence of a range of virulence factors result in a modest variation in the clinical manifestations and laboratory findings of leptospirosis [23]. Another study conducted in Malaysia demonstrates that women who experience an ectopic pregnancy are more likely to have tested positive for chlamydia than those who give birth at term [24]. Zheng et al. from Shanghai, China, investigate the bacterial communities and the prevalence of some primary pathogens in *Haemaphysalis* spp., the dominant species of ticks in Shanghai [25]. Chakraborty et al. analyze the prescriptions of patients suffering from diarrhea or acute respiratory infection in order to understand the prescription pattern among various categories of prescribers in two tertiary care centers in West Bengal, India. They reveal that irrational prescribing patterns prevail in tertiary care centers [26]. 

Cumulatively, the 26 articles collected in this issue provide readers with a broad overview of emerging diseases and foreground the advances made in our understanding of several domains related to the knowledge, surveillance and control of zoonotic diseases. We sincerely thank the authors, reviewers and the editorial staff members for their contributions to this Special Issue.
